# 2-Chloro-*N*-(4-methyl­phen­yl)benzamide

**DOI:** 10.1107/S1600536811041651

**Published:** 2011-10-12

**Authors:** Vinola Z. Rodrigues, Peter Herich, B. Thimme Gowda, Jozef Kožíšek

**Affiliations:** a Department of Chemistry, Mangalore University, Mangalagangotri 574 199, Mangalore, India; b Institute of Physical Chemistry and Chemical Physics, Slovak University of Technology, Radlinského 9, SK-812 37 Bratislava, Slovak Republic

## Abstract

In the title compound, C_14_H_12_ClNO, the *ortho*-Cl atom in the benzoyl ring is positioned *syn* to the C=O bond. The benzoyl and aniline benzene rings are tilted relative to each other by 82.8 (1)°. In the crystal, inter­molecular N—H⋯O hydrogen bonds link the mol­ecules into infinite chains running along the *c*-axis direction.

## Related literature

For the preparation of the title compound, see: Gowda *et al.* (2003 ▶). For studies on the effects of substituents on the structures and other aspects of *N*-(ar­yl)-amides, see: Bowes *et al.* (2003 ▶); Gowda *et al.* (2000 ▶); Saeed *et al.* (2010 ▶), on *N*-(ar­yl)-methane­sulfonamides, see: Gowda *et al.* (2007 ▶), on *N*-(ar­yl)-aryl­sulfonamides, see: Shetty & Gowda (2005 ▶) and on *N*-chloro-aryl­sulfonamides, see: Gowda & Shetty (2004 ▶).
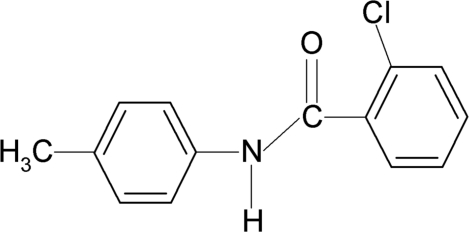


## Experimental

### 

#### Crystal data

C_14_H_12_ClNO
*M* *_r_* = 245.70
Monoclinic, 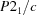

*a* = 20.2969 (14) Å
*b* = 7.1850 (5) Å
*c* = 8.8662 (5) Å
β = 93.750 (5)°
*V* = 1290.22 (15) Å^3^
*Z* = 4
Mo *K*α radiation
μ = 0.28 mm^−1^
*T* = 293 K
0.92 × 0.28 × 0.07 mm

#### Data collection

Oxford Diffraction Xcalibur Ruby Gemini diffractometer
Absorption correction: analytical [*CrysAlis RED* (Oxford Diffraction, 2009 ▶), based on expressions derived from Clark & Reid (1995 ▶)] *T*_min_ = 0.911, *T*_max_ = 0.981
21209 measured reflections
3594 independent reflections
1870 reflections with *I* > 2σ(*I*)
*R*_int_ = 0.038

#### Refinement

*R*[*F* ^2^ > 2σ(*F* ^2^)] = 0.057
*wR*(*F* ^2^) = 0.138
*S* = 1.03
3594 reflections
154 parameters
H-atom parameters constrained
Δρ_max_ = 0.35 e Å^−3^
Δρ_min_ = −0.42 e Å^−3^

### 

Data collection: *CrysAlis CCD* (Oxford Diffraction, 2009 ▶); cell refinement: *CrysAlis CCD*; data reduction: *CrysAlis RED* (Oxford Diffraction, 2009 ▶); program(s) used to solve structure: *SHELXS97* (Sheldrick, 2008 ▶); program(s) used to refine structure: *SHELXL97* (Sheldrick, 2008 ▶); molecular graphics: *DIAMOND* (Brandenburg, 2002 ▶); software used to prepare material for publication: *enCIFer* (Allen *et al.*, 2004 ▶).

## Supplementary Material

Crystal structure: contains datablock(s) I, global. DOI: 10.1107/S1600536811041651/bt5668sup1.cif

Structure factors: contains datablock(s) I. DOI: 10.1107/S1600536811041651/bt5668Isup2.hkl

Supplementary material file. DOI: 10.1107/S1600536811041651/bt5668Isup3.cml

Additional supplementary materials:  crystallographic information; 3D view; checkCIF report

## Figures and Tables

**Table 1:** Hydrogen-bond geometry (Å, °)

*D*—H⋯*A*	*D*—H	H⋯*A*	*D*⋯*A*	*D*—H⋯*A*
N1—H1*A*⋯O1^i^	0.86	2.01	2.847 (2)	165

Symmetry code: (i) 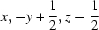.

**Fig. 1.**
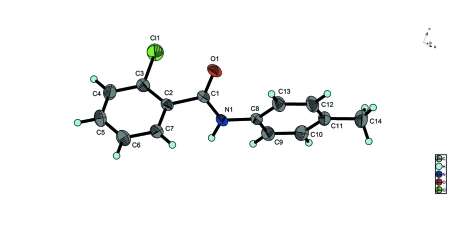
Molecular structure of the title compound showing the atom labelling scheme. Displacement ellipsoids are drawn at the 50% probability level. H atoms are represented as small spheres of arbitrary radii.

**Fig. 2.**
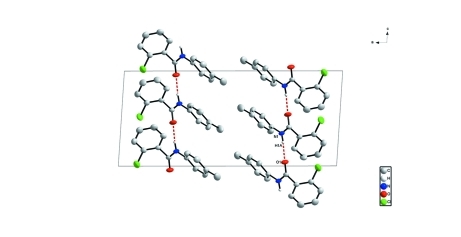
Part of the crystal structure of the title compound. Molecular chains are generated by N—H···O hydrogen bonds which are shown by dashed lines. H atoms not involved in intermolecular bonding have been omitted.

## supplementary crystallographic information

### Comment

The amide and sulfonamide moieties are the constituents of many biologically significant compounds. As part of our studies on the substituent effects on the structures and other aspects of *N*-(aryl)-amides (Bowes *et al.*, 2003; Gowda *et al.*, 2000; Saeed *et al.*, 2010), *N*-(aryl)-methanesulfonamides (Gowda *et al.*, 2007), *N*-(aryl)-arylsulfonamides (Shetty & Gowda, 2005) and *N*-chloro-arylsulfonamides (Gowda & Shetty, 2004), in the present work, the crystal structure of 2-chloro-*N*-(4-methylphenyl)benzamide (I) has been determined (Fig.1).

In (I), the *ortho*-Cl atom in the benzoyl ring is positioned *syn* to the C=O bond and *anti* to the N–H bond of the C—NH—C(O)—C segment.

The central amide group –NHCO– is tilted to the anilino ring with the C9—C8—N1—C1 and C13—C8—N1—C1 torsion angles of -144.4 (2)° and 35.6 (3)°. The C3—C2—C1—N1 and C7—C2—C1—N1 torsion angles are -118.8 (2)° and 64.0 (3)°, respectively, while the C3—C2—C1—O1 and C7—C2—C1—O1 torsion angles are 61.8 (3)° and -115.4 (2)°, respectively. But the C2—C1—N1—C8 and C8—N1—C1—O1 torsion angles are 178.0 (2)° and -2.7 (3)°, respectively.

In the crystal structure, intermolecular N—H···O hydrogen bonds link the molecules into infinite chains running along the *c*-axis. Part of the crystal structure is shown in Fig. 2.

### Experimental

The title compound was prepared according to the method described by Gowda *et al.* (2003). The purity of the compound was checked by determining its melting point. It was characterized by recording its infrared and NMR spectra. Rod like colourless single crystals of the title compound used in X-ray diffraction studies were obtained by slow evaporation of an ethanol solution of the compound (0.5 g in 30 ml of ethanol) at room temperature.

### Refinement

All hydrogen atoms were placed in calculated positions with C–H distances of 0.93Å (C-aromatic), 0.96Å (C-methyl), 0.86Å (N-H) and constrained to ride on their parent atoms. The *U*_iso_(H) values were set at 1.2 *U*_eq_ (C-aromatic, N) and 1.5 *U*_eq_(C-methyl).

### Crystal data

**Table:**

C_14_H_12_ClNO	*F*(000) = 512
*M**_r_* = 245.70	*D*_x_ = 1.265 Mg m^−^^3^
Monoclinic, *P*2_1_/*c*	Mo *K*α radiation, λ = 0.71073 Å
Hall symbol: -P 2ybc	Cell parameters from 3776 reflections
*a* = 20.2969 (14) Å	θ = 3.5–29.5°
*b* = 7.1850 (5) Å	µ = 0.28 mm^−^^1^
*c* = 8.8662 (5) Å	*T* = 293 K
β = 93.750 (5)°	Rod, colorless
*V* = 1290.22 (15) Å^3^	0.92 × 0.28 × 0.07 mm
*Z* = 4	

### Data collection

**Table:**

Oxford Diffraction Xcalibur Ruby Gemini diffractometer	3594 independent reflections
Radiation source: Enhance (Mo) X-ray Source	1870 reflections with *I* > 2σ(*I*)
graphite	*R*_int_ = 0.038
Detector resolution: 10.4340 pixels mm^-1^	θ_max_ = 29.5°, θ_min_ = 3.5°
ω scans	*h* = −28→28
Absorption correction: analytical [*CrysAlis RED* (Oxford Diffraction, 2009), based on expressions derived from Clark & Reid (1995)]	*k* = −9→9
*T*_min_ = 0.911, *T*_max_ = 0.981	*l* = −12→12
21209 measured reflections	

### Refinement

**Table:**

Refinement on *F*^2^	Primary atom site location: structure-invariant direct methods
Least-squares matrix: full	Secondary atom site location: difference Fourier map
*R*[*F*^2^ > 2σ(*F*^2^)] = 0.057	Hydrogen site location: inferred from neighbouring sites
*wR*(*F*^2^) = 0.138	H-atom parameters constrained
*S* = 1.03	*w* = 1/[σ^2^(*F*_o_^2^) + (0.0457*P*)^2^ + 0.4284*P*] where *P* = (*F*_o_^2^ + 2*F*_c_^2^)/3
3594 reflections	(Δ/σ)_max_ < 0.001
154 parameters	Δρ_max_ = 0.35 e Å^−^^3^
0 restraints	Δρ_min_ = −0.42 e Å^−^^3^

### Special details

**Table:**

Experimental. CrysAlis RED (Oxford Diffraction, 2009) Analytical numeric absorption correction using a multifaceted crystal model based on expressions derived (Clark & Reid, 1995).
Geometry. All e.s.d.'s (except the e.s.d. in the dihedral angle between two l.s. planes) are estimated using the full covariance matrix. The cell e.s.d.'s are taken into account individually in the estimation of e.s.d.'s in distances, angles and torsion angles; correlations between e.s.d.'s in cell parameters are only used when they are defined by crystal symmetry. An approximate (isotropic) treatment of cell e.s.d.'s is used for estimating e.s.d.'s involving l.s. planes.
Refinement. Refinement of *F*^2^ against ALL reflections. The weighted *R*-factor *wR* and goodness of fit *S* are based on *F*^2^, conventional *R*-factors *R* are based on *F*, with *F* set to zero for negative *F*^2^. The threshold expression of *F*^2^ > σ(*F*^2^) is used only for calculating *R*-factors(gt) *etc*. and is not relevant to the choice of reflections for refinement. *R*-factors based on *F*^2^ are statistically about twice as large as those based on *F*, and *R*- factors based on ALL data will be even larger.

### Fractional atomic coordinates and isotropic or equivalent isotropic displacement parameters (Å^2^)

**Table:**

	*x*	*y*	*z*	*U*_iso_*/*U*_eq_	
C1	0.22887 (10)	0.1405 (3)	0.4095 (2)	0.0490 (5)	
C2	0.17841 (10)	0.0438 (3)	0.30648 (19)	0.0481 (5)	
C3	0.11200 (11)	0.0438 (3)	0.3330 (2)	0.0568 (5)	
C4	0.06674 (12)	−0.0529 (4)	0.2409 (3)	0.0719 (6)	
H4A	0.0221	−0.0500	0.2589	0.086*	
C5	0.08826 (14)	−0.1534 (4)	0.1225 (3)	0.0820 (8)	
H5A	0.0581	−0.2215	0.0614	0.098*	
C6	0.15356 (14)	−0.1548 (4)	0.0931 (3)	0.0808 (8)	
H6A	0.1675	−0.2227	0.0118	0.097*	
C7	0.19841 (11)	−0.0560 (3)	0.1836 (2)	0.0616 (6)	
H7A	0.2427	−0.0559	0.1624	0.074*	
C8	0.31056 (9)	0.3950 (3)	0.4145 (2)	0.0524 (5)	
C9	0.31260 (12)	0.5767 (4)	0.3668 (3)	0.0693 (6)	
H9A	0.2826	0.6178	0.2901	0.083*	
C10	0.35880 (13)	0.6989 (4)	0.4316 (3)	0.0797 (7)	
H10A	0.3590	0.8218	0.3987	0.096*	
C11	0.40448 (12)	0.6430 (4)	0.5435 (3)	0.0757 (7)	
C12	0.40141 (11)	0.4610 (5)	0.5898 (3)	0.0802 (8)	
H12A	0.4315	0.4201	0.6664	0.096*	
C13	0.35533 (11)	0.3354 (4)	0.5273 (2)	0.0668 (6)	
H13A	0.3548	0.2130	0.5612	0.080*	
C14	0.45614 (15)	0.7752 (5)	0.6112 (3)	0.1150 (12)	
H14C	0.4833	0.7117	0.6876	0.138*	
H14B	0.4349	0.8791	0.6556	0.138*	
H14A	0.4831	0.8187	0.5335	0.138*	
N1	0.26167 (8)	0.2768 (2)	0.34391 (17)	0.0544 (4)	
H1A	0.2521	0.2947	0.2491	0.065*	
O1	0.23840 (8)	0.0938 (2)	0.54154 (14)	0.0692 (5)	
Cl1	0.08462 (3)	0.17186 (12)	0.48200 (8)	0.0953 (3)	

### Atomic displacement parameters (Å^2^)

**Table:**

	*U*^11^	*U*^22^	*U*^33^	*U*^12^	*U*^13^	*U*^23^
C1	0.0552 (11)	0.0531 (12)	0.0382 (10)	0.0015 (10)	−0.0008 (8)	−0.0014 (9)
C2	0.0600 (12)	0.0441 (11)	0.0395 (10)	−0.0023 (9)	−0.0016 (8)	0.0020 (9)
C3	0.0613 (13)	0.0547 (13)	0.0540 (11)	0.0004 (10)	0.0018 (10)	−0.0008 (10)
C4	0.0613 (14)	0.0739 (16)	0.0796 (16)	−0.0134 (12)	−0.0017 (12)	−0.0015 (14)
C5	0.0859 (19)	0.0788 (18)	0.0796 (17)	−0.0274 (15)	−0.0074 (14)	−0.0225 (14)
C6	0.096 (2)	0.0765 (18)	0.0701 (15)	−0.0153 (15)	0.0047 (14)	−0.0292 (14)
C7	0.0690 (14)	0.0618 (14)	0.0541 (12)	−0.0077 (11)	0.0048 (10)	−0.0105 (11)
C8	0.0491 (11)	0.0684 (15)	0.0396 (10)	−0.0058 (10)	0.0023 (8)	−0.0047 (10)
C9	0.0732 (15)	0.0747 (17)	0.0586 (13)	−0.0149 (13)	−0.0072 (11)	0.0063 (12)
C10	0.0863 (18)	0.0782 (18)	0.0751 (16)	−0.0268 (15)	0.0082 (14)	−0.0070 (14)
C11	0.0594 (14)	0.104 (2)	0.0648 (15)	−0.0233 (14)	0.0090 (12)	−0.0228 (15)
C12	0.0530 (14)	0.116 (2)	0.0688 (15)	0.0004 (15)	−0.0146 (11)	−0.0135 (16)
C13	0.0573 (13)	0.0780 (16)	0.0631 (13)	0.0035 (12)	−0.0111 (11)	−0.0031 (12)
C14	0.085 (2)	0.156 (3)	0.105 (2)	−0.051 (2)	0.0105 (17)	−0.046 (2)
N1	0.0606 (10)	0.0670 (11)	0.0341 (8)	−0.0105 (9)	−0.0069 (7)	0.0021 (8)
O1	0.0919 (11)	0.0781 (11)	0.0360 (7)	−0.0142 (9)	−0.0084 (7)	0.0082 (7)
Cl1	0.0758 (5)	0.1171 (6)	0.0947 (5)	0.0072 (4)	0.0185 (4)	−0.0381 (4)

### Geometric parameters (Å, °)

**Table:**

C1—O1	1.221 (2)	C8—C13	1.375 (3)
C1—N1	1.339 (2)	C8—N1	1.420 (2)
C1—C2	1.498 (3)	C9—C10	1.382 (3)
C2—C3	1.383 (3)	C9—H9A	0.9300
C2—C7	1.387 (3)	C10—C11	1.373 (4)
C3—C4	1.377 (3)	C10—H10A	0.9300
C3—Cl1	1.731 (2)	C11—C12	1.373 (4)
C4—C5	1.369 (3)	C11—C14	1.510 (3)
C4—H4A	0.9300	C12—C13	1.389 (3)
C5—C6	1.368 (4)	C12—H12A	0.9300
C5—H5A	0.9300	C13—H13A	0.9300
C6—C7	1.372 (3)	C14—H14C	0.9600
C6—H6A	0.9300	C14—H14B	0.9600
C7—H7A	0.9300	C14—H14A	0.9600
C8—C9	1.374 (3)	N1—H1A	0.8600
			
O1—C1—N1	124.43 (18)	C8—C9—C10	120.6 (2)
O1—C1—C2	121.11 (18)	C8—C9—H9A	119.7
N1—C1—C2	114.45 (15)	C10—C9—H9A	119.7
C3—C2—C7	118.16 (18)	C11—C10—C9	121.5 (3)
C3—C2—C1	122.10 (17)	C11—C10—H10A	119.3
C7—C2—C1	119.68 (18)	C9—C10—H10A	119.3
C4—C3—C2	121.3 (2)	C10—C11—C12	116.9 (2)
C4—C3—Cl1	119.06 (18)	C10—C11—C14	121.5 (3)
C2—C3—Cl1	119.65 (16)	C12—C11—C14	121.6 (3)
C5—C4—C3	119.1 (2)	C11—C12—C13	122.8 (2)
C5—C4—H4A	120.4	C11—C12—H12A	118.6
C3—C4—H4A	120.4	C13—C12—H12A	118.6
C6—C5—C4	120.8 (2)	C8—C13—C12	118.9 (3)
C6—C5—H5A	119.6	C8—C13—H13A	120.5
C4—C5—H5A	119.6	C12—C13—H13A	120.5
C5—C6—C7	119.9 (2)	C11—C14—H14C	109.5
C5—C6—H6A	120.0	C11—C14—H14B	109.5
C7—C6—H6A	120.0	H14C—C14—H14B	109.5
C6—C7—C2	120.7 (2)	C11—C14—H14A	109.5
C6—C7—H7A	119.7	H14C—C14—H14A	109.5
C2—C7—H7A	119.7	H14B—C14—H14A	109.5
C9—C8—C13	119.2 (2)	C1—N1—C8	126.81 (15)
C9—C8—N1	117.82 (18)	C1—N1—H1A	116.6
C13—C8—N1	122.9 (2)	C8—N1—H1A	116.6
			
O1—C1—C2—C3	61.8 (3)	C13—C8—C9—C10	−0.4 (3)
N1—C1—C2—C3	−118.8 (2)	N1—C8—C9—C10	179.6 (2)
O1—C1—C2—C7	−115.4 (2)	C8—C9—C10—C11	0.9 (4)
N1—C1—C2—C7	63.9 (2)	C9—C10—C11—C12	−1.1 (4)
C7—C2—C3—C4	0.3 (3)	C9—C10—C11—C14	178.3 (3)
C1—C2—C3—C4	−177.0 (2)	C10—C11—C12—C13	0.8 (4)
C7—C2—C3—Cl1	−178.22 (16)	C14—C11—C12—C13	−178.6 (2)
C1—C2—C3—Cl1	4.5 (3)	C9—C8—C13—C12	0.0 (3)
C2—C3—C4—C5	1.1 (4)	N1—C8—C13—C12	−180.0 (2)
Cl1—C3—C4—C5	179.6 (2)	C11—C12—C13—C8	−0.2 (4)
C3—C4—C5—C6	−1.6 (4)	O1—C1—N1—C8	−2.7 (3)
C4—C5—C6—C7	0.6 (4)	C2—C1—N1—C8	178.00 (18)
C5—C6—C7—C2	0.9 (4)	C9—C8—N1—C1	−144.4 (2)
C3—C2—C7—C6	−1.3 (3)	C13—C8—N1—C1	35.6 (3)
C1—C2—C7—C6	176.0 (2)		

### Hydrogen-bond geometry (Å, °)

**Table:**

*D*—H···*A*	*D*—H	H···*A*	*D*···*A*	*D*—H···*A*
N1—H1A···O1^i^	0.86	2.01	2.847 (2)	165.

Symmetry codes: (i) *x*, −*y*+1/2, *z*−1/2.
